# Investigation of the Amathillopsidae (Amphipoda, Crustacea), including the description of a new species, reveals a clinging lifestyle in the deep sea worldwide

**DOI:** 10.3897/zookeys.1031.62391

**Published:** 2021-04-14

**Authors:** Anne-Nina Lörz, Tammy Horton

**Affiliations:** 1 Center of Naturkunde, Universität Hamburg, Martin-Luther King Platz 3, 20146 Hamburg, Germany Universität Hamburg Hamburg Germany; 2 National Oceanography Centre, Southampton, European Way, Southampton SO14 3ZH, UK National Oceanography Centre Southampton United Kingdom

**Keywords:** Benthic, Porcupine Abyssal Plain, Remotely Operated Vehicle (ROV), worldwide distribution

## Abstract

Amathillopsidae is a widely distributed, but rarely sampled family of deep-sea amphipods. During a recent expedition to the North Atlantic, specimens were filmed clinging to a polychaete tube in situ at abyssal depths by a Remote Operated Vehicle and then sampled for further study. The species was new to science and is described in detail herein. A barcode sequence is provided. Further investigations of photographic and video records revealed the genus *Amathillopsis* to be more widely distributed, both geographically and bathymetrically, than indicated by current literature records, and that these species occur at abyssal depths in all oceans. Specimens of *Amathillopsis* are reported clinging to a variety of different organisms whose erect structures provide the means to raise these charismatic deep-sea predators above the seafloor facilitating feeding opportunities.

## Introduction

During the third expedition of the Icelandic Genetics & Evolution (IceAGE) project on the RV ‘Sonne’ (Cruise SO267) to the North Atlantic from June to July 2020, large numbers of amphipod crustaceans were collected using a variety of methods. Using the Remotely Operated Vehicle (ROV) KIEL 6000, an interesting amathillopsid amphipod was observed sitting as a pair clinging to an onuphid worm tube at 4600 m depth at the Porcupine Abyssal Plain. The amathillopsid species was successfully sampled and proved to be new to science. A single specimen of the new species, collected at the Porcupine Abyssal Plain Sustained Observatory site at 4844 m, was also found within the Discovery Collections at the National Oceanography Centre, Southampton, UK. This new species, amongst the deepest confirmed record of the genus, is described herein.

Lowry (2006) included the parepimeriids and the genus *Cleonardopsis* K.H. Barnard, 1916 in the family Amathillopsidae and created three subfamilies: Amathillopsinae, Parepimeriinae and Cleonardopsinae. The new species of *Amathillopsis* described herein is classified within the Amathillopsinae.

Three species of *Amathillopsis* and one species of *Cleonardopsis* have been reported from the North Atlantic to date: *Amathillopsis affinis* Miers, 1881; *Amathillopsis spinigera* Heller, 1875; *Amathillopsis atlantica* Chevreux, 1908; and a probable new species of *Cleonardopsis* which was first reported from off the coast of eastern Greenland by Stephensen (1944) as *Cleonardopsis carinata* K.H. Barnard,1916, and may be the same species as the *Cleonardopsis* sp. sequenced by Jażdżewska et al. (2018).

The Amathillopsidae are rarely collected, and very little is known of their biology and ecology. Most studies of the family relate to the description of new species based on material from a single or very few specimens, from a single locality.

In recent years, the increased use of ROVs to capture high-resolution footage of deep-sea ecosystems has provided an opportunity for the study of poorly known and rarely captured organisms (Macreadie et al. 2018). This is certainly the case with the Amathillopsidae. We provide collated records of ROV footage of a number of as yet unidentified species of *Amathillopsis*, from the deep sea, worldwide; thus confirming that despite the paucity of records and specimens, the genus is cosmopolitan and relatively common.

## Materials and methods

### Collection methods and locations

During the IceAGE 3 expedition on the RV ‘Sonne’, the ROV KIEL 6000 sampled the Porcupine Abyssal Plain at station 133-4 (49°47.969'N, 015°12.975'E, 4622 m, 20 July 2020), via photo and video transects, as well as physical sampling. The specimens of the new species of *Amathillopsis* were initially photographed and filmed in situ, after which attempts were made to use the ROV suction to collect them. When this failed the specimens were scooped up by the ROV operator arm using a net and then placed into a sampling box.

Once on board, the single specimen collected was immediately photographed and then placed in RNA*later*. The left first pleopod was then dissected and placed in a separate tube to be used for DNA extraction. The whole specimen and the dissected pleopod sample were then both transferred to the -20 °C freezer for later study.

The type localities and holotype materials of all known species of *Amathillopsis* were collated to aid in future studies of the genus (Table 1). Additional photographic records of other *Amathillopsis* specimens were collated from a variety of sources (Table 3), along with associated metadata, allowing the mapping of specimen localities.

**Table 1.** Type localities of all described species (and subspecies) of *Amathillopsis* and *Cleonardopsis*. The type locality of *Amathillopleustes alticoxa* is included as this likely represents a different species from *Cleonardopsis carinata*, with which it is currently synonymised. All localities are taken from original descriptions. Coordinates of localities for *A. annectens*, *A. pacifica*, and *C. carinata* are inferred from the verbatim type locality.

Species	Verbatim latitude and verbatim longitude	Decimal latitude and longitude	Depth (m)	Geographic locality
*Amathillopsis affinis*	79°55'N, 51°E	80.5667, 54.7833	unknown	Arctic Ocean
*Amathillopsis annectens*	SE point Santa Catalina Island, 3.2 miles	33.2735, -118.2705	611–1097	North Pacific, California
*Amathillopsis atlantica*	39°11'N, 30°24'W	39.1833, -30.4000	1600–1919	North Atlantic, Azores
*Amathillopsis australis*	12°8'S, 145°10'E	-12.3333, 145.1667	2560	Coral Sea, Celebes Sea, Arafura Sea
*Amathillopsis charlottae*	66°33.10'S, 68°41.90'W	-66.5528, -68.7083	607	Antarctic Peninsula
*Amathillopsis comorensis*	12°14.4'S, 46°41.6'E	-11.6520, 43.3726	2500	Indian Ocean, Comoros
*Amathillopsis grevei*	-44.3, 166.7667	-44.3, 166.7667	3580	Tasman Sea
*Amathillopsis pacifica margo*	23°59.5'N, 113°11.9'W	23.9847, -113.1858	3479–3515	North Pacific, Baja California
*Amathillopsis pacifica*	Southern Basin Okhotsk Sea	52.8736, 149.3658	2850	Okhotsk Sea, North Pacific
*Amathillopsis roroi*	-60.61833, -54.93167	-60.3710, -54.9317	3213	Antarctic Peninsula
*Amathillopsis septemdentata*	13°46'S, 47°33'E	-13.7667, 47.5500	1490–1600	Indian Ocean, Nosy-Be, Madagascar
*Amathillopsis spinigera*	79°15'N, 60°E	77.8750, 20.9752	240	Arctic Ocean
*Amathillopsis takahashiae*	31.43889, 131.67333	31.4389, 131.6733	528	North Pacific, Japan
*Amathillopsis inkenae* sp. nov.	50.0525, -15.470833	50.0525, -15.4708	4622	North Atlantic, Porcupine Abyssal Plain
*Cleonardopsis carinata*	36 miles NNE Cape Point	-34.3567, 18.4968	1189	South Africa, South Atlantic
*Amathillopleustes alticoxa*	2°40'S, 128°37'.5E	-2.9358, 128.6181	835	Ceram Sea, Indonesia

### Taxonomic methods

The adult male holotype specimen (ZMH K 60236) was photographed in situ by the ROV KIEL 6000, photographed on board by a Nikon D5 camera with an objective Nikon AF-S Mikro-Nikkor 105 mm 1:2.8, and dissected appendages were photographed using a Keyence 7000 microscope. A video of the *Amathillopsis* in situ can be found in the Suppl. material 1.

Initial observations and photographs were made on board of the RV ‘Sonne’.

The pencil drawings were conducted using a LeicaM125 and an Olympus BX53. Pencil drawings were scanned and inked digitally using Adobe Illustrator and a WACOM digitiser tablet (Coleman 2003, 2009). Some setae are omitted from the illustrations for clarity. Type material is deposited in the Zoological Museum Hamburg (**ZMH**) and the Natural History Museum, London (**NHMUK**).

### Genetic methods

Isolation of DNA was performed on board using the NucleoSpin tissue extraction kit from MACHEREY-NAGEL GmbH & Co. KG according to the manufacturer’s protocol. A fragment of the COI gene (ca. 670 bp fragment) was amplified using primers LCO1490-JJ CHACWAAYCATAAAGATATYGG Forward (Astrin and Stüben 2008) and HCO2198-JJ AWACTTCVGGRTGVCCAAARAATCA Reverse (Astrin and Stüben 2008).

The PCR reaction mixes were prepared to a final volume of 25 µl containing 12.5 µl AccuStart II PCR ToughMix (Quanta Bio), 0.5 µl of each primer (10 pmol/µl), 9.5 µl dH2O and 2 µl template DNA. PCR settings for amplifying CO1 sequences consisted of initial denaturing of 4 min at 95 °C, 5 cycles of 45 s at 95 °C, 90 s at 45 °C, 60 s at 72 °C, following 35 cycles of 45 s at 95 °C, 60 s at 51 °C, 60 s at 72 °C, and final extension 3 min at 72 °C. PCR products were purified using the Exonuclease-I/Shrimp Alkaline Phosphatase (Thermo Fisher) method and were sequenced at Macrogen Inc. Europe. Sequences were edited using Geneious 9.1.8 resulting in a sequence of length of 626 bp excluding primers. Relevant voucher information, taxonomic classifications and sequences are deposited in BOLD.

Setal and mouthpart classifications follow Watling (1989) and Lowry and Stoddart (1992, 1993, 1995).

The following abbreviations have been used:

**A** antenna;

**E** epimeron;

**Ep** epistome;

**G** gnathopod;

**H** Head;

**LL** lower lip;

**Md** mandible;

**Mx** maxilla;

**Mxp** maxilliped;

**P** pereopod;

**T** telson;

**U** uropod;

**UL** upper lip.

## Systematics

### Order Amphipoda Latreille, 1816

**Suborder Amphilochidea Boeck, 1871**

**Infraorder Amphilochida Boeck, 1871**

**Parvorder Amphilochidira Boeck, 1871**

**Superfamily Iphimedioidea Boeck, 1871**

**Family Amathillopsidae Pirlot, 1934**

#### Subfamily Amathillopsinae Pirlot, 1934

##### Amathillopsis

Taxon classificationAnimaliaAmphipodaAmathillopsidae

Heller, 1875

3858272E-D7D9-54CE-84AF-1829E15B635D

Amathillopsis Heller, 1875: 35. – Stebbing 1906: 384. – Gurjanova 1955: 209 (key). – J.L. Barnard 1969: 394. – J.L. Barnard and Karaman 1991: 390.
Acanthopleustes Holmes, 1908: 533 (Acanthopleustes annectens Holmes, 1908 by original designation).

###### Type species.

*Amathillopsis spinigera* Heller, 1875 (by original designation).

###### Diagnosis

**(after Lowry 2006). *Head*.** Deeper than long; lateral cephalic lobe subquadrate, truncated apically; anteroventral margin straight, anteroventral margin moderately recessed, anteroventral margin moderately excavate; rostrum short or moderate length; eyes present (round or ovoid) or absent. Body smooth, or dorsally carinate. Antenna 1 subequal in length or longer than antenna 2; peduncle with sparse slender setae; peduncular article 1 shorter than or subequal to article 2; article 2 longer than article 3; article 3 shorter than article 1; accessory flagellum short or minute, 1- or 2-articulate; calceoli present. Antenna 2 medium length; peduncle with sparse slender setae or none; flagellum shorter than or as long as peduncle.

***Pereon*.** Coxae 1–4 longer than broad, overlapping, coxae 1–3 or coxae 1–4 ventrally acute. Coxae 1–3 similar in size or progressively larger. Gnathopod 1 subchelate; carpus shorter than or subequal to propodus; propodus with or without peg-like robust setae along palmar margin. Gnathopod 2 subchelate; coxa smaller than but not hidden by coxa 3 or subequal to but not hidden by coxa 3; carpus short, shorter than propodus. Pereopods: some or none prehensile. Pereopod 4 coxa ventrally acute, with or without small posteroventral lobe. Pereopod 5 coxa equilobate, with posteroventral lobe or with acute posterodistal lobe; basis slightly expanded or linear. Pereopod 6 subequal in length to, or longer than pereopod 7; basis slightly expanded or linear. Pereopod 7 shorter than or subequal in length to pereopod 5; basis slightly expanded or linear.

***Pleon*.** Urosomite 1 carinate, urosomites 1–2 carinate or urosomites not carinate. Uropods 1–2 apices of rami without robust setae. Telson notched, emarginate or entire; dorsal or lateral robust setae absent; apical robust setae absent.

###### Remarks.

*Amathillopsis* is the type genus of the family Amathillopsidae and the genus has a cosmopolitan distribution (Wakabara and Serejo 1999). It currently contains 12 species, five of which are known from the Pacific, *A. annectens* (Holmes, 1908), *A. australis* Stebbing, 1883, *A. grevei* J.L. Barnard, 1961, *A. takahashiae* Tomikawa & Mawatari, 2006 and *A. pacifica* Gurjanova, 1955 (and the sub species *A. pacifica margo* J.L. Barnard, 1967),; two from the Antarctic, *A. roroi* Coleman & Coleman, 2008 and *A. charlottae* Coleman, 1998; two from the Indian Ocean, *A. septemdentata* Ledoyer, 1978 and *A. comorensis* Ledoyer, 1986; and three from the North Atlantic and Arctic, *A. affinis* Miers, 1881, *A. spinigera* Heller, 1875 and *A. atlantica* Chevreux, 1908. *Amathillopsis* is a deep-sea specialist, with the shallowest records of the large species, *A. spinigera*, coming from colder Arctic waters at 248 m. All other species are found at depths of 500 to 3580 m. The new species, *A. inkenae*, described here, provides the deepest confirmed records of an *Amathillopsis* to date, at 4622 m and 4844 m. Table 1 summarises the known species in the genus along with the type locality and depth.

###### Species.

*Amathillopsis affinis* Miers, 1881, *A. annectens* (Holmes, 1908), *A. atlantica* Chevreux, 1908, *A. australis* Stebbing, 1883, *A. charlottae* Coleman, 1998, *A. comorensis* Ledoyer, 1986, *A. grevei* J.L. Barnard, 1961, *A. pacifica* Gurjanova, 1955, *A. pacifica margo* J.L. Barnard, 1967, *A. roroi* Coleman & Coleman, 2008, *A. septemdentata* Ledoyer, 1978, *A. spinigera* Heller, 1875, *A. takahashiae* Tomikawa & Mawatari, 2006.

##### Amathillopsis inkenae sp. nov.

Taxon classificationAnimaliaAmphipodaAmathillopsidae

5AFE79F0-9DF7-5A16-A29E-0EC806AA2ECC

http://zoobank.org/19555683-216D-434B-866C-A5B710BDA2A6

**Figure 1.**
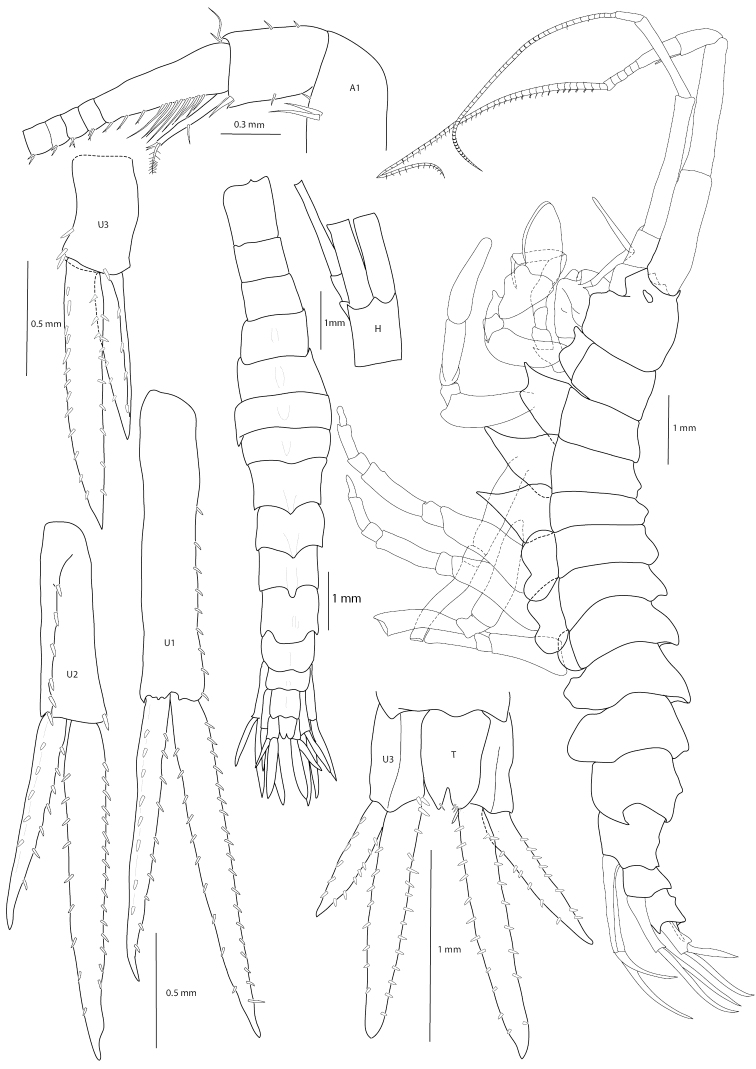
*Amathillopsis inkenae* sp. nov. Holotype: ZMH K-60236, male, 9.4 mm. Habitus lateral and dorsal views, head, antenna 1, uropods 1–3, and telson.

**Figure 2.**
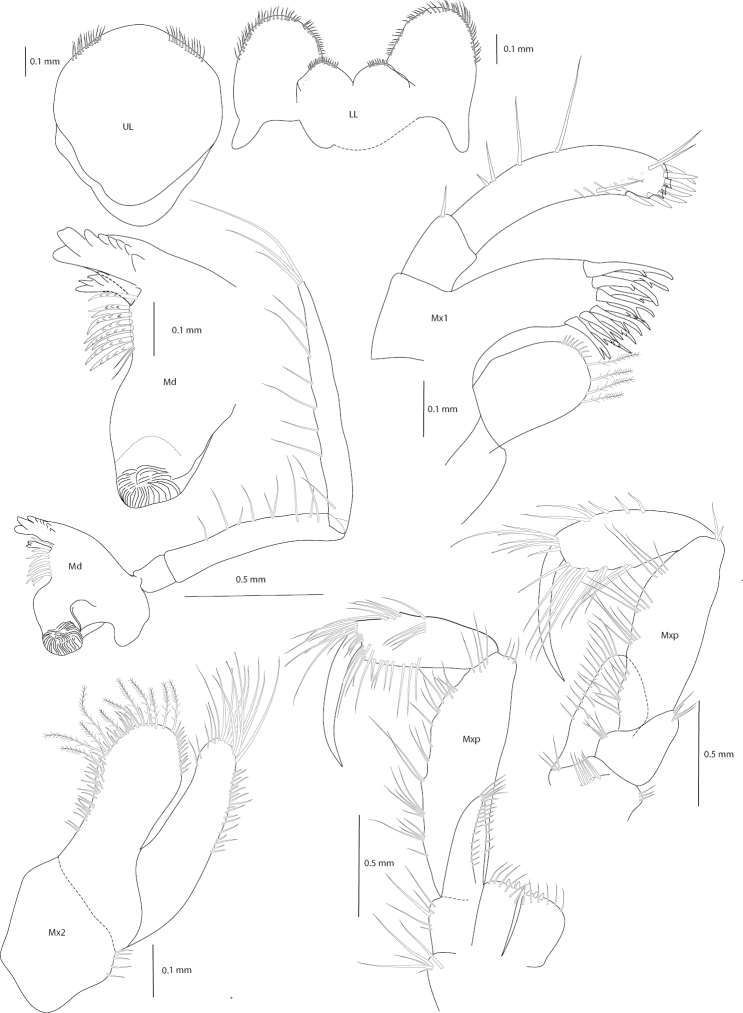
*Amathillopsis inkenae* sp. nov., Holotype: ZMH K-60236, male, 9.4 mm. Upper lip, Lower lip, Maxilliped (left palp external rotation is an artefact of the mounting), maxillae 1, 2, and mandible.

**Figure 3.**
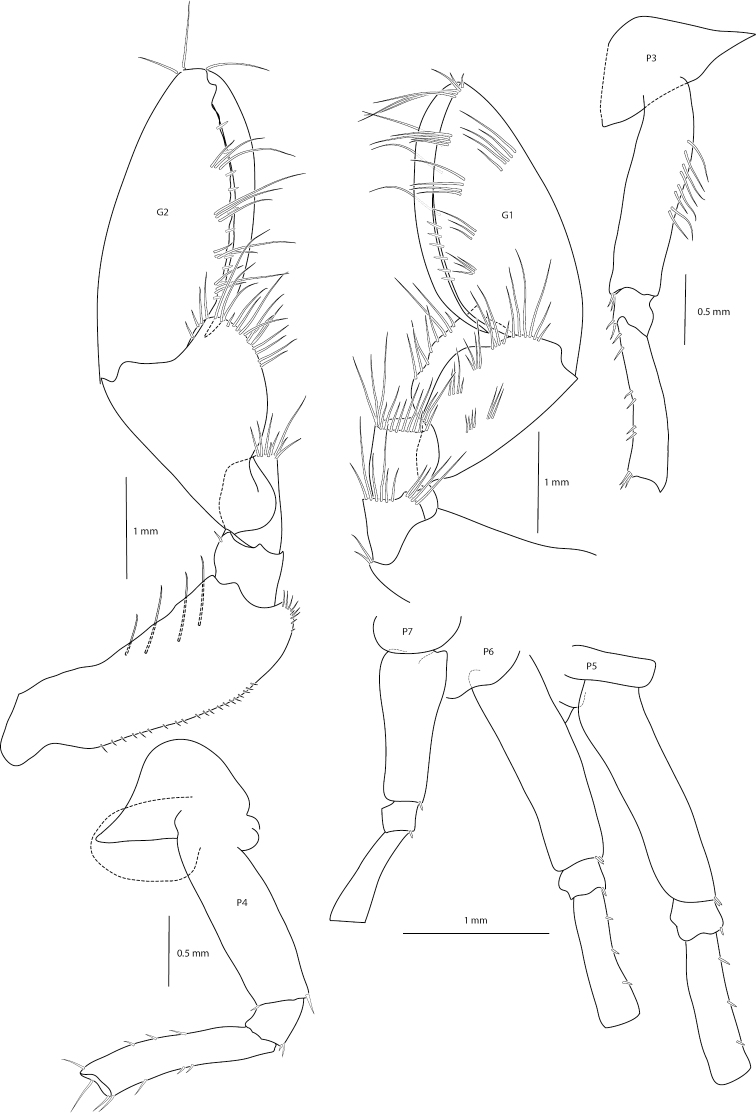
*Amathillopsis inkenae* sp. nov., Holotype: ZMH K-60236, male, 9.4 mm. Gnathopods 1, 2, and pereopods 3–7.

**Figure 4.**
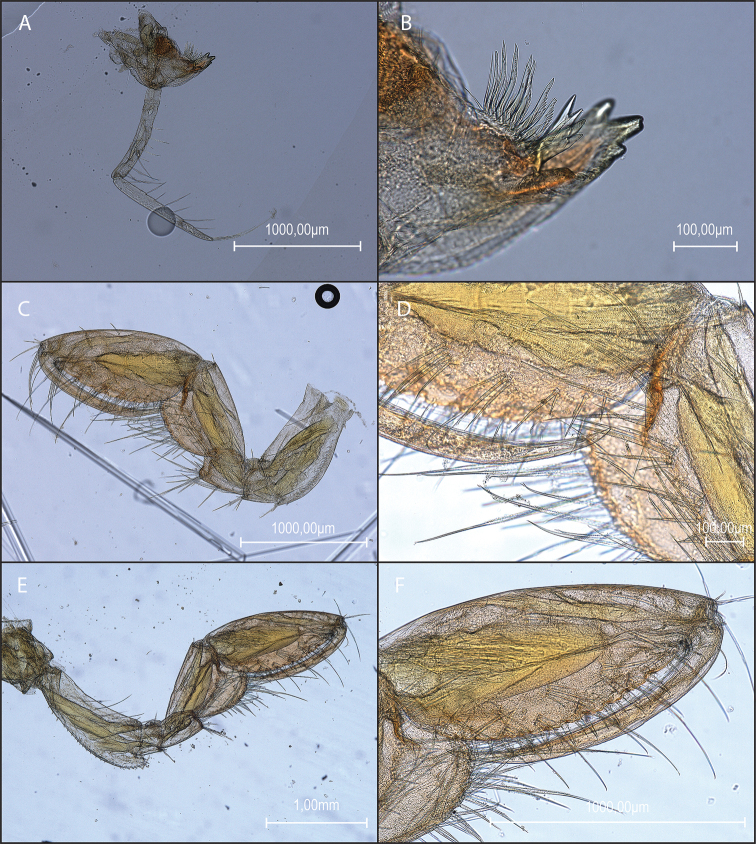
*Amathillopsis inkenae* sp. nov., Holotype: ZMH K-60236, male, 9.4 mm **A** mandible **B** mandible incisor detail **C** gnathopod 1 **D** gnathopod 1 detail **E** gnathopod 2 **F** gnathopod 2 palm detail.

**Figure 5.**
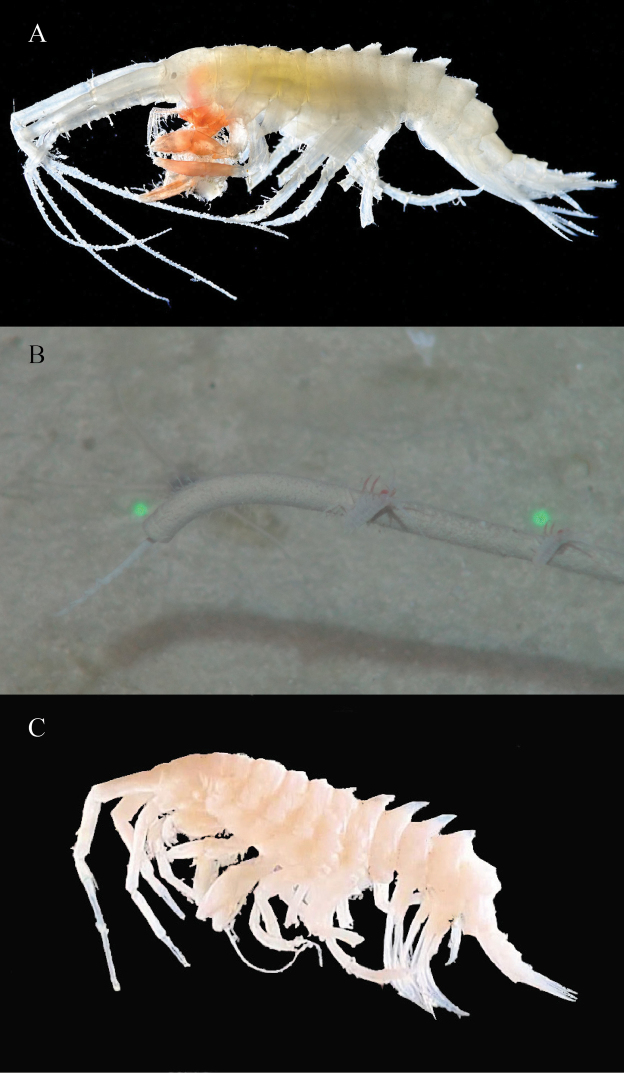
*Amathillopsis inkenae* sp. nov., Holotype: ZMH K-60236, male, 9.4 mm **A** habitus, fresh specimen photograph (S. Zankl) **B** in situ image clinging to a polychaete tube via ROV KIEL 6000. In situ video can be found in Suppl. material 1**C***Amathillopsis inkenae* sp. nov., paratype NHMUK 2021.66, male, 14 mm. Habitus, preserved specimen (photograph T. Horton).

Figures 1 , 2 , 3 , 4 , 5

###### Type material.

***Holotype*:** North East Atlantic • Male, 9.4 mm; Porcupine Abyssal Plain; 49°47.969'N, 015°12.975'E, 4622 m; 20 July 2020; RV ‘Sonne’ cruise 267, station 133–4, gear ROV KIEL 6000; ZMH K-60236. ***Paratype***: North East Atlantic • Male, 14 mm; Porcupine Abyssal Plain; 48°58.201'N, 016°53.297'W to 48°55.316'N, 016°49.452'W, 4834–4844 m; 06 June 2018; RRS ‘James Cook’ Cruise 165, station JC165#064, Otter Trawl Semi-Balloon 14; NHMUK 2021.66.

###### Type locality.

Porcupine Abyssal Plain, 4622 m, 49°47.969'N, 015°12.975'E, RV ‘Sonne’ cruise 267, station 133–4, gear ROV KIEL 6000.

###### Diagnosis.

Pereonites 3 and 4 with small, rounded mid-dorsal projections. Pereonites 5–7 mid-dorsal projections, small, rounded, increasing in size. Pleonites 1 and 2 mid-dorsal projections small, rounded, reduced to dorsal hump on pleonite 3. Urosomite 1 mid-dorsal projection absent, urosomites 2 and 3 carinate, urosomite 3 with a small mid-dorsal process. Gnathopod 2 posterodistal basis lobe developed. Strong, acute tooth on posterodistal corner of epimeron 3. Telson cleft.

###### Description.

Male holotype 9.4 mm: Head slightly shorter than pereonites 1 and 2 combined, rostrum very short, pointed, lateral cephalic lobe quadrate, eyes present, pigmented, strongly white in fresh specimen. Pereonites 1 and 2 indistinctly keeled dorsally; pereonite 3–5 with short mid-dorsal processes; pereonites 6 and 7 each with short, weakly posteriorly curved mid-dorsal process. Pleonites 1 and 2 each with short weakly posteriorly curved mid-dorsal process; pleonite 3 with low, mid-dorsal rounded process. Epimeral plates 1 and 2 with ventral margin rounded, posteroventral corner rounded; epimeral plate 3 with ventral margin curved and posteroventral corner produced into an acute tooth. Urosomite 1 lacking dorsal armature, urosomites 2–3 dorsally carinate, each with short weakly posteriorly curved mid-dorsal process, urosomite 3 with a small mid-dorsal process. Antenna 1 long, as long as body length, with peduncular articles 1, 2, and 3 in length ratio of 1.0: 1.1: 0.4. Article 1 longer than head length; accessory flagellum uni-articulate, not spine-like; primary flagellum consisting of 64 articles, article 1 long, as long as articles 2–7 combined. Antenna 2 0.8 × as long as antenna 1; peduncular article 3 reaching to mid length of peduncular article 1 of antenna 1; peduncular article 4 long, 1.7 × as long as peduncular article 5, flagellum approximately the same length, as long as peduncle, 54-articulate.

***Mouthparts*.** Upper lip with weakly convex apical margin, bearing two groups of setae. Lower lip with outer lobes broad, setulose; inner lobes indistinct, fused. Mandibles with left incisors bearing eight teeth, left *lacinia mobilis* with four teeth; accessory setal row with nine setae, some bearing a row of minute protuberances. Molar developed, triturative. Palp articles 1, 2, and 3 in length ratio of 1.0: 5.0: 7.1, article 1 lacking setae, article 2 with marginal and submarginal setae, and article 3 with six marginal and three terminal setae. Maxilla 1 with inner plate ovate and bearing four plumose setae; outer plate rectangular, with 11 serrate, robust setae; palp two-articulate, longer than outer plate, terminally with seven long robust setae. Maxilla 2 inner plate slightly broader than outer plate, bearing row of long plumose setae. Maxilliped, inner plate reaching base of palp, with three robust nodular setae on the distomedial margin, distolateral margin with apical robust setae; outer plate exceeding distal margin of palp article 1. Maxillipedal palp long, raptorial, four-articulate; article 2 and 3 heavily setose and widened medially; dactylus as long as article 3.

***Pereon*.** Coxae 1 and 2 with acute processes projecting anteroventrally. Coxa 3 subtriangular, Coxa 4 rhomboid, both with acute processes projecting anteroventrally. Coxae 5 and 6 wider than long, bilobate. Coxa 7 small, rounded. Gnathopod 1 subchelate, basis posterior margin with row of robust setae, posterodistal lobe absent; ischium and merus short; carpus 0.68 × as long as propodus, ventral lobe broad, concave, allowing propodus to retract; propodus stout, tapering distally, with four groups of robust setae, palmar margin with long and short robust setae; dactylus as long as palmar margin, sickle-like. Gnathopod 2 subchelate, basis with posterodistal lobe present, posterior margin with row of robust setae; carpus 0.67 × as long as propodus, ventral lobe broad, concave, allowing propodus to retract; propodus stout, tapering distally, with four groups of robust setae, palmar margin with long and short robust setae; dactylus as long as palmar margin, sickle-like. Pereopod 3 basis with row of robust setae along weakly convex posterior margin, ischium short, as long as wide; merus margins subparallel with slight anterior curvature. Pereopod 4 similar to pereopod 3. Pereopods 5–7 anterior and posterior margins of basis sub-parallel, linear, posterior lobe lacking; ischium short, as long as wide; merus margins subparallel with slight anterior curvature. Carpus, propodus and dactylus missing from pereopods 3–7.

***Uropods*.** Uropod 1 long, peduncle length 0.88 × inner ramus; medial margin of peduncle with robust setae, inner ramus, lateral and medial margins with robust setae, outer ramus 0.88 × as long as inner, lateral and medial margins with robust setae. Uropod 2 with peduncle length 0.57 × inner ramus, lateral margin with robust setae, dorsomedial margin with one robust seta distally; inner ramus, lateral and medial margins with robust setae; outer ramus 0.64 × inner, lateral and medial margins with robust setae. Uropod 3 peduncle length 0.74 × inner ramus; dorsomedial margin of peduncle with three robust setae distally; inner ramus with lateral and medial margins bearing robust setae, outer ramus 0.64 × as long as inner, lateral, and medial margins with robust setae. Telson length 1.44 × width, cleft 22%. Each lobe bearing terminal setae.

**Paratype male**, 14 mm: As for holotype except the dorsal processes are more pronounced and acute on pereonites 5–7 and pleonites 1 and 2 (Fig. 5c).

###### Etymology.

The name is dedicated to Dr. Inken Suck, the pilot who flew the ROV and sampled the specimen, to honour her dedication to deep-sea biology.

###### Remarks.

In live condition, *Amathillopsis inkenae* sp. nov. has a white coloured body and antennae, the last three segments of both gnathopods as well as the mouthparts are red. Eyes are clearly visible, solid white, in live and fresh condition, but fade after a few days of fixation. Care should be taken in use of the relative sizes of the dorsal processes as these are likely to vary ontogenetically, as for the two specimens available here, where the larger male paratype has more pronounced, acute processes than the smaller male holotype. This is also likely to occur in other species in the genus. The specimens reported by Wakabara and Serejo (1999) as *A. atlantica* are likely to belong to a new species, and the authors point out that the mid-dorsal processes and the telson show some variation. Other characters, such as the reduced lobes on the basis of the gnathopods, also indicate that this is probably a new taxon.

*Amathillopsis inkenae* sp. nov. differs from known species of *Amathillopsis* by the characters listed in Table 2. *Amathillopsis inkenae* sp. nov. is most similar to *Amathillopsis comorensis* Ledoyer, 1986, which was collected in the Indian Ocean near the Comoros Islands at 2500 m. The new species has a similarly cleft telson, and a similar development of the dorsal processes and of the lobe on the basis of gnathopod 2 only. *Amathillopsis inkenae* differs from *A. comorensis* in having carination on urosomites 2 and 3, with posteriorly directed dorsal processes (lacking in *A. comorensis*), the stronger acute tooth on epimeron 3 posterodistal corner (smaller on *A. comorensis*), the curved article 2 of the mandible palp (straight in *A. comorensis*), and the medially widened articles 2 and 3 of the maxilliped palp (subparallel sided in *A. comorensis*).

**Table 2.** Morphological tabulation of characters for separating known species of *Amathillopsis*.

Character	*A. inkenae* sp. nov.	*A. affinis*	*A. annectens*	*A. atlantica*	*A. australis*	*A. charlottae*	*A. comorensis*	*A. grevei*	*A. pacifica*	*A. p. margo*	*A. roroi*	*A. septemdentata*	*A. spinigera*	*A. takahashiae*
**Pereonites 1–4 mid-dorsal projections**	small, rounded on 3 & 4	strong, acute on 1–4	small, rounded on 2–4	absent	keeled on 1–4	keeled on 2–4	small, rounded on 2–4	absent	small, rounded on 2–4	small, rounded on 2–4	absent	absent	strong, acute on 1–4	absent
**Pereonites 5–7 mid-dorsal projections**	small, rounded, increasing in size on 5–7	strong, acute on 5–7	medium, acute, increasing in size on 5–7	strong, acute, increasing in size on 5–7	strong, acute, increasing in size on 5–7	strong, acute, increasing in size on 5–7	strong, acute, increasing in size on 5–7	medium, acute, increasing in size on 5–7	strong, acute, increasing in size on 5–7	strong, acute, increasing in size on 5–7	absent but trace of keel on 6–7	strong, acute, increasing in size on 5–7	strong, acute on 5–7	rounded hump on 5, acute on 6–7
**Pleonites 1–3 mid-dorsal projections**	1–2 small, rounded, 3 reduced to dorsal hump	strong, acute on 1–2, 3 small	medium, acute, 1–3, smaller on 3	strong, acute on 1–3	strong, acute on 1–3	strong, acute on 1–2, slightly smaller on 3	medium acute on 1–2 , 3 with tiny upright process	medium, acute, 1–3, decreasing	strong, acute on 1–2, smaller on 3	strong, acute on 1–2, smaller on 3	1–2 short, acute, 3 small upright process	strong, acute on 1–3	strong, acute on 1–3	strong, acute on 1–2, smaller on 3
**Urosomite 1 mid-dorsal projection**	absent	unknown	absent	absent	absent	absent	absent	absent	absent	absent	absent	present	present	absent
**Gnathopod posterodistal basis lobe**	developed on G2 only	unknown	absent	well-developed on G1 and G2	developed on G2 only	developed on G1 and G2	developed on G2 only	slightly developed on G2 only	well-developed	well-developed	small	small	small	small
**Mandible palp article 3: article 2 length**	1.4	unknown	1.2	unknown	1.5	1.1	1.4	unknown	1.4	1.4	1	1.5	0.9	0.9
**Telson**	cleft	emarginate	entire	emarginate	emarginate	entire	cleft	emarginate	emarginate	emarginate	emarginate	entire	emarginate	emarginate (with dorsal keel)
**Antenna 1 Accessory Flagellum**	uniarticulate, ordinary	uniarticulate, ordinary	uniarticulate, ordinary	uniarticulate, spine-like, straight	uniarticulate, spine-like, curved	uniarticulate, ordinary	uniarticulate, spine-like	uniarticulate, ordinary	uniarticulate, spine-like, straight	uniarticulate, spine-like, straight	uniarticulate, ordinary	uniarticulate, spine-like, straight	bi-articulate, ordinary	uniarticulate

The barcode of *Amathillopsis inkenae* sp. nov. is deposited in BOLD:AEF9286 and GenBank MW726208.

###### Depth range.

4622–4844 m.

###### Distribution.

Only known from the North East Atlantic Ocean, Porcupine Abyssal Plain, between 4622–4844 m.

## Discussion

We have described a new species of *Amathillopsis* collected from abyssal depths and differentiated this new species from the known species found globally. Only *A. grevei*, *A. roroi*, and *A. pacifica* have been collected at abyssal depths; all other *Amathillopsis* species were collected shallower than 2000 m. However, photographs and video captured by ROVs are now able to show that the genus is relatively common at bathyal and abyssal depths. *Amathillopsis* species have now been observed by ROVs and other camera systems on a number of occasions, clinging in pairs (and occasionally in larger numbers), to a tubular or stalk-like structure erected from soft substrate, and also on corals attached to hard substrates. We have collated these records and present them alongside the type localities of known *Amathillopsis* species (Table 3, Fig. 7).

**Table 3.** Locality data for collated photographic records of specimens of *Amathillopsis*. DISCOL = DIS-turbance and re-COL-onization experiment; APEI = Areas of Particular Environmental Interest; TOML = Tonga Offshore Mining Limited.

Geographic Locality	Latitude and longitude	Depth (m)	Date (dd/mm/yyyy)	Publication/credit
**North Atlantic, Porcupine Abyssal Plain**	50.0525, -15.4708	4622	20/07/2020	This study
**Kiribati (east of the Line Island Group)**	5.9903, -156.7402	4660	02/08/2015	https://doi.org/10.3389/fmars.2019.00605
**Kiribati (west of the Line Island Group)**	2.5704, -162.2069	5111	30/07/2015	https://doi.org/10.3389/fmars.2019.00605
**Kiribati (east of the Phoenix Islands Group)**	-0.0001, -170.9988	5559	27/07/2015	https://doi.org/10.3389/fmars.2019.00605
**Kiribati (east of the Line Island Group)**	5.9725, -156.7832	4653	02/08/2015	https://doi.org/10.3389/fmars.2019.00605
**Eastern Clarion Clipperton Zone (TOML-C)**	15.2734, -129.6792	5002	02/09/2015	https://doi.org/10.1016/j.pocean.2020.102405
**Eastern Clarion Clipperton Zone (APEI-6)**	17.3400, -122.9007	4005	07/05/2015	https://doi.org/10.1016/j.pocean.2018.11.003
**Eastern Clarion Clipperton Zone (APEI-6)**	17.3575, -122.9053	4013	07/05/2015	https://doi.org/10.1016/j.pocean.2018.11.003
**Eastern Clarion Clipperton Zone (APEI-6)**	17.2421, -122.8223	4239	10/05/2015	https://doi.org/10.1016/j.pocean.2018.11.003
**Peru Basin – DISCOL site**	-7.0736, -88.4653	4130	24/03/2017	https://doi.org/10.1038/s41598-019-44492-w
**Peru Basin – DISCOL site**	-7.1258, -88.4568	4160	24/03/2017	https://doi.org/10.1038/s41598-019-44492-w
**Peru Basin – DISCOL site**	-7.0801, -88.4678	4133	24/03/2017	https://doi.org/10.1038/s41598-019-44492-w
**Peru Basin – DISCOL site**	-7.1252, -88.4506	4149	15/09/2015	courtesy of GEOMAR
**Peru Basin – DISCOL site**	-7.0898, -88.4463	4140	13/09/2015	courtesy of GEOMAR
**New Zealand, Abyssal basin between Three Kings & Colville Ridges**	-30.9908, 177.5010	4159	01/02/2017	courtesy of GEOMAR
**Northern Mariana Islands, Southern Marianas, Fina Nagu Volcanic Chain**	12.7956, 143.7862	2629	27/04/2016	courtesy of NOAA Office of Ocean Exploration and Research
**Northern Mariana Islands, Marianas Trench Marine National Monument**	21.5679, 145.5185	3300	29/06/2016	courtesy of NOAA Office of Ocean Exploration and Research
**Northern Mariana Islands, Marianas Trench Marine National Monument**	20.7234, 145.0618	1909	01/07/2016	courtesy of NOAA Office of Ocean Exploration and Research
**Pacific Remote Islands Marine National Monument, northeast of Kingman Reef**	6.4178, -162.2202	1930	14/05/2017	courtesy of NOAA Office of Ocean Exploration and Research
**North West Pacific, Emperor Seamount Chain, Suiko Seamount**	44.5561, 170.4798	2252	08/08/2019	Schmidt Ocean Institute, courtesy of NOAA Office of Ocean Exploration and Research
**North West Pacific, Emperor Seamount Chain, Yomei Seamount**	42.4313, 170.4371	1495	09/08/2019	Schmidt Ocean Institute, courtesy of NOAA Office of Ocean Exploration and Research
**North West Pacific, Emperor Seamount Chain, Yomei Seamount**	42.4313, 170.4377	1493	09/08/2019	Schmidt Ocean Institute, courtesy of NOAA Office of Ocean Exploration and Research
**North West Pacific, Emperor Seamount Chain, Yomei Seamount**	42.4318, 170.4357	1479	09/08/2019	Schmidt Ocean Institute, courtesy of NOAA Office of Ocean Exploration and Research
**North West Pacific, Emperor Seamount Chain, Yomei Seamount**	42.4319, 170.4354	1472	09/08/2019	Schmidt Ocean Institute, courtesy of NOAA Office of Ocean Exploration and Research
**North West Pacific, Emperor Seamount Chain, Yomei Seamount**	42.4402, 170.4381	1336	09/08/2019	Schmidt Ocean Institute, courtesy of NOAA Office of Ocean Exploration and Research
**North West Pacific, Emperor Seamount Chain, Yomei Seamount**	42.4320, 170.4350	1470	09/08/2019	Schmidt Ocean Institute, courtesy of NOAA Office of Ocean Exploration and Research
**North West Pacific, Emperor Seamount Chain, Nintoku Seamount**	40.7519, 170.5925	1490	12/08/2019	Schmidt Ocean Institute, courtesy of NOAA Office of Ocean Exploration and Research
**Sea of Okhotsk, Bussol Strait**	46.9426, 151.0836	3299	22/07/2015	https://doi.org/10.1016/j.dsr2.2018.05.022
**Aleutian Islands**	52.4981, -174.9232	2947	27/07/2004	ROV JASON, courtesy of Les Watling

**Figure 6.**
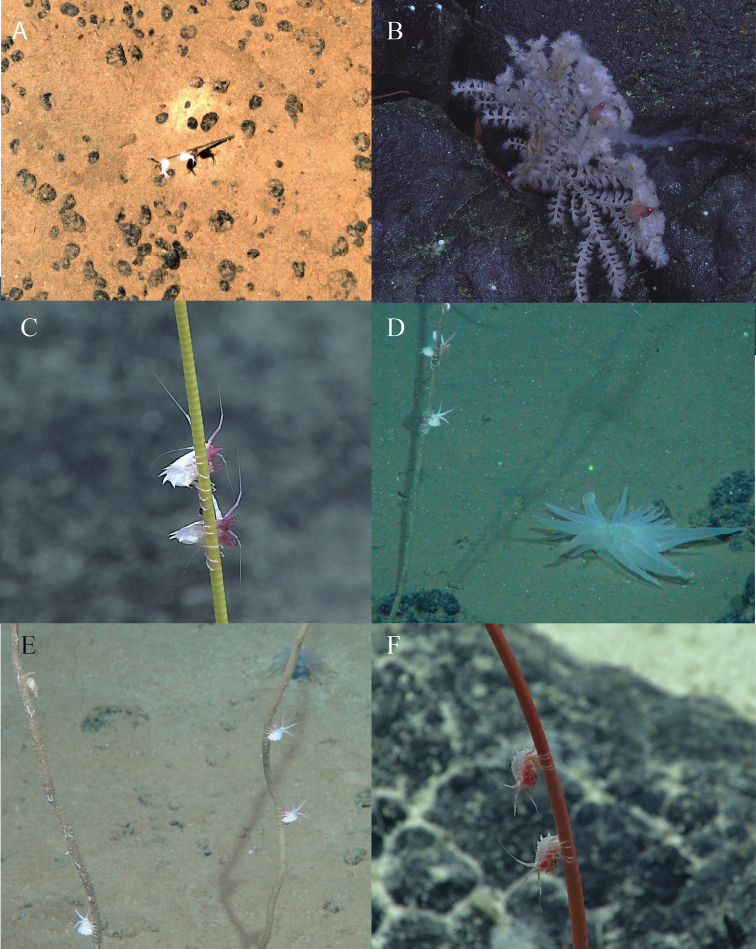
A selection of photographic records of specimens of *Amathillopsis*: **A** Eastern Clarion Clipperton Zone, APEI-6, 4013 m **B** Emperor Seamount Chain, Yomei Seamount, 1470 m **C** Northern Mariana Islands, Fina Nagu Volcanic Chain, 2629 m **D** Peru Basin, DISCOL site, 4149 m **E** New Zealand, Abyssal basin, 4160 m **F** northeast of Kingman Reef, 1930 m. See Table 3 for detailed information.

**Figure 7.**
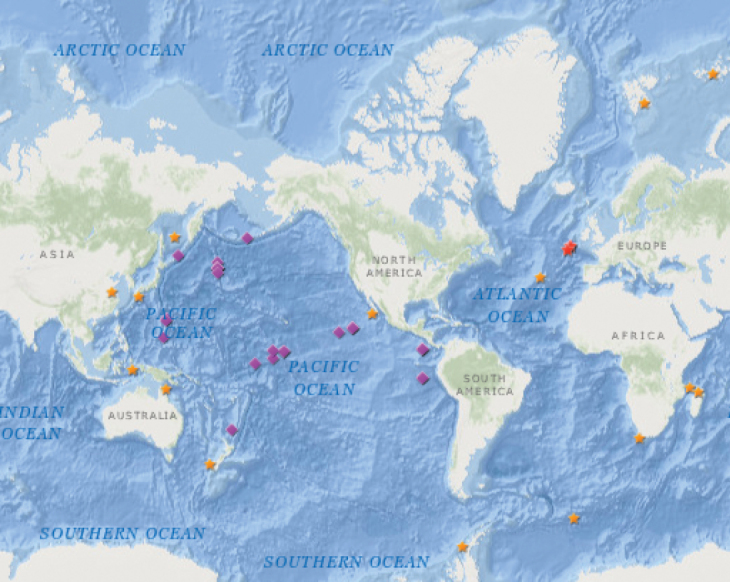
Map showing the type localities of known species of *Amathillopsis* (orange stars), the location of photograph records of *Amathillopsis* species (purple diamonds), and type localities of *Amathillopsis inkenae* sp. nov. (red stars). The types of the two specimens of Cleonardopsinae are included. See Table 3 for detailed locality information.

All photographic records of *Amathillopsis* collated here are from the Pacific (Fig. 7, purple diamonds), while *Amathillopsis* species are distributed globally (Fig. 7, orange stars). The NOAA Office of Ocean Exploration and Research Benthic Deepwater Animal Identification Guide includes a number of images of amphipods of the genus *Amathillopsis* from the central Pacific at the Northern Mariana Islands (Fig. 6c) and the Pacific Remote Islands Marine National Monument, northeast of Kingman Reef (Fig. 6f). There a number of images of pairs of *Amathillopsis* from abyssal depths in the Eastern Clarion Clipperton Zone (Fig. 6a) from Kiribati waters, and from bathyal depths of the northwest Pacific at the Emperor Seamounts (Fig. 6b) which were collected during AUV and ROV surveys of these areas.

The ROV KIEL 6000 captured images of pairs of *Amathillopsis* clinging to sponges below 4000 m during the DISCOL expedition on RV ‘Sonne’ to the southeast Pacific in 2015 (Fig. 6d) and to the abyssal plains North of New Zealand (southwest Pacific) in 2017 (Fig. 6e). We have also included in Table 3 and Fig. 7 a recently published record of a specimen of *Amathillopsis*. The specimen shown in Brandt et al. (2018: fig. 6a) is probably a specimen of *A. pacifica*, since it conforms to the illustrations of that species by Gurjanova (1955), and it was collected from a similar geographic locality and depth. We have not made any further attempts to identify the specimens in these images to species, since it is likely that there are new species involved and therefore specimens will be needed for more detailed analyses. It is remarkable that the type localities and the photo localities (see Fig. 7) are so different. While dragged (towed) gear has coincidently sampled occasional specimens of *Amathillopsis*, the advent of technology has resulted in numerous additional records of these animals in situ, work which has been largely focussed in the Pacific Ocean. *Amathillopsis inkenae* sp. nov. was observed with two individuals clinging to a polychaete worm tube (video, Suppl. material 1) at 4622 m depth on the Porcupine Abyssal Plain. During the observation time of 20 minutes, the amphipods did not alter their position on the tube. The tube belongs to a polychaete worm of the family Onuphidae (Alexandra Kerbl, pers. comm., August 2020). Unfortunately, we only managed to retrieve one of the two specimens. Nevertheless, this is the first confirmed record of an *Amathillopsis* species observed in situ which has been subsequently collected and deposited in a natural history collection. Stills camera and video technology have developed in recent years to a level that enables high resolution images at bathyal and abyssal depths. Until now, the habits of species of *Amathillopsis* could only be postulated, on the basis of their possession of posterior pereopods adapted to a clinging lifestyle. The functional morphology enabling the gripping of thin structures is expressed by the strongly curved pereopodal dactyli and the pereopods being flexed backwards. These adaptations were described by McCloske (1970) as well-adapted for grasping, in reference to *Dulichia* clinging onto sea urchin spines. While clinging behaviour is known from a number of amphipod families in shallow waters, such as Caprellidae, Podoceridae, and Dulichiidae (e.g., Takeuchi and Hirano 1995; Guerra-García et al. 2002), it is only now that we are able to report that amathillopsids express this behaviour at bathyal and abyssal depths of all oceans. We assume the stem they cling to is simply a means to expose them higher in the water column for feeding. *Amathillopsis inkenae* was found on a worm tube, whereas other *Amathillopsis* species have been photographed clinging to a variety of other organism structures, including sponges and corals.

Based on the raptorial structure of the mouthparts and gnathopods we assume *Amathillopsis* to be predators (or micropredators), capturing their prey, such as zooplankton or small suprabenthic crustaceans from the water column. The red colour of gnathopods and mouthparts may result from the consumption of carotinoids from prey. Amathillopsids have never been caught in baited traps, and therefore we exclude the possibility of them being scavengers. Also remarkable are the well-developed eyes of *Amathillopsis* specimens living below 3000 m. It is probable that they rely on bioluminescence as communication, either for catching prey, avoiding predators or finding mating partners.

## Supplementary Material

XML Treatment for Amathillopsis

XML Treatment for Amathillopsis inkenae

## Supplementary materials

Supplementary material 1 In situ video Data type mp4. video file Explanation note *Amathillopsis inkenae* sp. nov., clinging onto a polychaete tube in 4622 m, Porcupine Abyssal Plain, filmed by the ROV KIEL 6000 during the RV ‘Sonne’ expedition 267, station 133-4. https://binary.pensoft.net/file/531626This dataset is made available under the Open Database License (http://opendatacommons.org/licenses/odbl/1.0/). The Open Database License (ODbL) is a license agreement intended to allow users to freely share, modify, and use this Dataset while maintaining this same freedom for others, provided that the original source and author(s) are credited.Anne-Nina Lörz, Tammy Horton
